# Rapid isolation of respiring skeletal muscle mitochondria using nitrogen cavitation

**DOI:** 10.3389/fphys.2023.1114595

**Published:** 2023-03-07

**Authors:** Awais Z. Younis, Gareth G. Lavery, Mark Christian, Craig L. Doig

**Affiliations:** Department of Biosciences, School of Science and Technology, Nottingham Trent University, Nottingham, United Kingdom

**Keywords:** mitochondria, cavitation, skeletal muscle, protocol, methodolody

## Abstract

Methods of isolating mitochondria commonly utilise mechanical force and shear stress to homogenize tissue followed by purification by multiple rounds of ultracentrifugation. Existing protocols can be time-consuming with some physically impairing integrity of the sensitive mitochondrial double membrane. Here, we describe a method for the recovery of intact, respiring mitochondria from murine skeletal muscle tissue and cell lines using nitrogen cavitation. This protocol results in high-yield, pure and respiring mitochondria without the need for purification gradients or ultracentrifugation. The protocol takes under an hour and requires limited specialised equipment. Our methodology is successful in extracting mitochondria of both cell extracts and skeletal muscle tissue. This represents an improved yield in comparison to many of the existing methods. Western blotting and electron microscopy demonstrate the enrichment of mitochondria with their ultrastructure well-preserved and an absence of contamination from cytoplasmic or nuclear fractions. Using respirometry analysis we show that mitochondria extracted from murine skeletal muscle cell lines (C2C12) and *tibialis anterior* tissue have an appropriate respiratory control ratio. These measures are indicative of healthy coupled mitochondria. Our method successfully demonstrates the rapid isolation of functional mitochondria and will benefit researchers studying mitochondrial bioenergetics as well as providing greater throughput and application for time-sensitive assays.

## Highlights

• Pure, respiring mitochondria in under an hour without complex purification gradients.
• Effective mitochondrial isolation from skeletal muscle cells and tissue.
• Potential use to those working with *in vivo* primary endpoints that have downstream *in vitro* mitochondrial evaluation.

## Introduction

Required for locomotion, respiration, and storage of nutrients, skeletal muscle depots are densely packed with mitochondria. As such, optimal mitochondrial function is fundamental to both cellular behaviour and preservation of whole-body energy homeostasis.

As appreciation of tissue-specific mitochondrial biology advances, there is a growing need to develop alternative methodological approaches to rapidly isolate and evaluate mitochondrial phenotype. However, to examine respiratory function, isolation from whole-cell constituents must be both efficient and robust. Currently, this can be achieved using a variety of techniques such as differential centrifugation (Drissi et al., 2013), affinity purification (Dunham et al., 2012) and free-flow electrophoresis/field-flow fractionation (Islinger et al., 2010). However, there are limitations to these methods including reduced purity and yield. Moreover, certain procedures require costly reagents (Sims and Anderson, 2008; Franko et al., 2013). Reproducible lysis of cellular membranes between samples is also important, particularly given the resilient nature of skeletal muscle cells (Franzini-Armstrong and Engel, 2012; Liao et al., 2020). Commonly, this is resolved through mechanical homogenisation or the application of shear stress. Whilst these are effective, they can compromise organelle integrity and produce compartment leakage. This is evidenced by a recent review (Liao et al., 2020) suggesting that mitochondria isolated using homogenisation show reduced integrity. Distinct methods of mitochondrial isolation also have specific requirements of time, technical capability, and specialist equipment, variables that contribute to differences in recovered purity and yield.

An ideal method should provide time-efficient mitochondrial isolation free from major contaminants and produce reproducible Respiratory Control Ratio (RCR) values consistent with an established understanding of mitochondrial function. Here we apply nitrogen cavitation to cultured skeletal muscle cells and murine tissue, a procedure in which nitrogen gas is diffused into cells under pressure and when released causes lysis of cell membranes for subsequent mitochondrial isolation (Gottlieb and Adachi, 2000). This coupled with a rapid differential centrifugation process allowed recovery of mitochondria in high yield, purity and suitable for high-resolution respiratory studies. Variation in RCR is low with ±0.2 and 0.8 SEM in isolated mitochondria from tissue and cells respectively. This protocol would benefit those studying mitochondria in skeletal muscle for high-resolution respiratory studies as well as for isolating mitochondria for proteome analysis.

## Methodology

### Animals

Mice (males) were purchase from Charles River, United Kingdom (B6j), they were group housed in standard temperature (22°C) and humidity-controlled environment with 12 h light 12 h dark cycle. Mice had *ad libitum* access to normal chow and water. Mice were sacrificed at 8 weeks of age using cervical dislocation. Skeletal muscle [*tibialis anterior* (TA)] was remove and placed in ice cold sucrose buffer (10 mM HEPES, pH 7.5, 70 mM sucrose, 200 mM mannitol, 1 mM EGTA, 1% protease inhibitors and 1% phosphatase inhibitors) for nitrogen cavitation and mitochondrial respirometry. All animal experiments were conducted in accordance with United Kingdom Home Office regulations, United Kingdom Animals (Scientific Procedures) Act 1986.

### Cell culture and tissue collection

For cells, C2C12 murine myoblasts (ATCC) in a T175 flask were maintained in high glucose- Dulbecco’s Minimal Eagle’s Medium (DMEM) supplemented with 10% (v/v) foetal bovine serum, 1% L-glutamine (v/v) (Life Technologies, Paisley, United Kingdom), and 1% (v/v) penicillin-streptomycin (Life Technologies) in a humidified atmosphere with 5% CO_2_ at 37°C. Once at 80% confluence, cells were incubated in DMEM supplemented with 2% (v/v) heat-inactivated horse serum (Life Technologies) for 5 days to facilitate myocyte differentiation with media being replaced every 48 h. Cells were used between passages 18–28. Skeletal muscle tissue (*tibialis anterior*) was freshly dissected from C57/BL6J and placed in ice cold sucrose extraction buffer.

### Mitochondrial isolation

**FIGURE 1:**
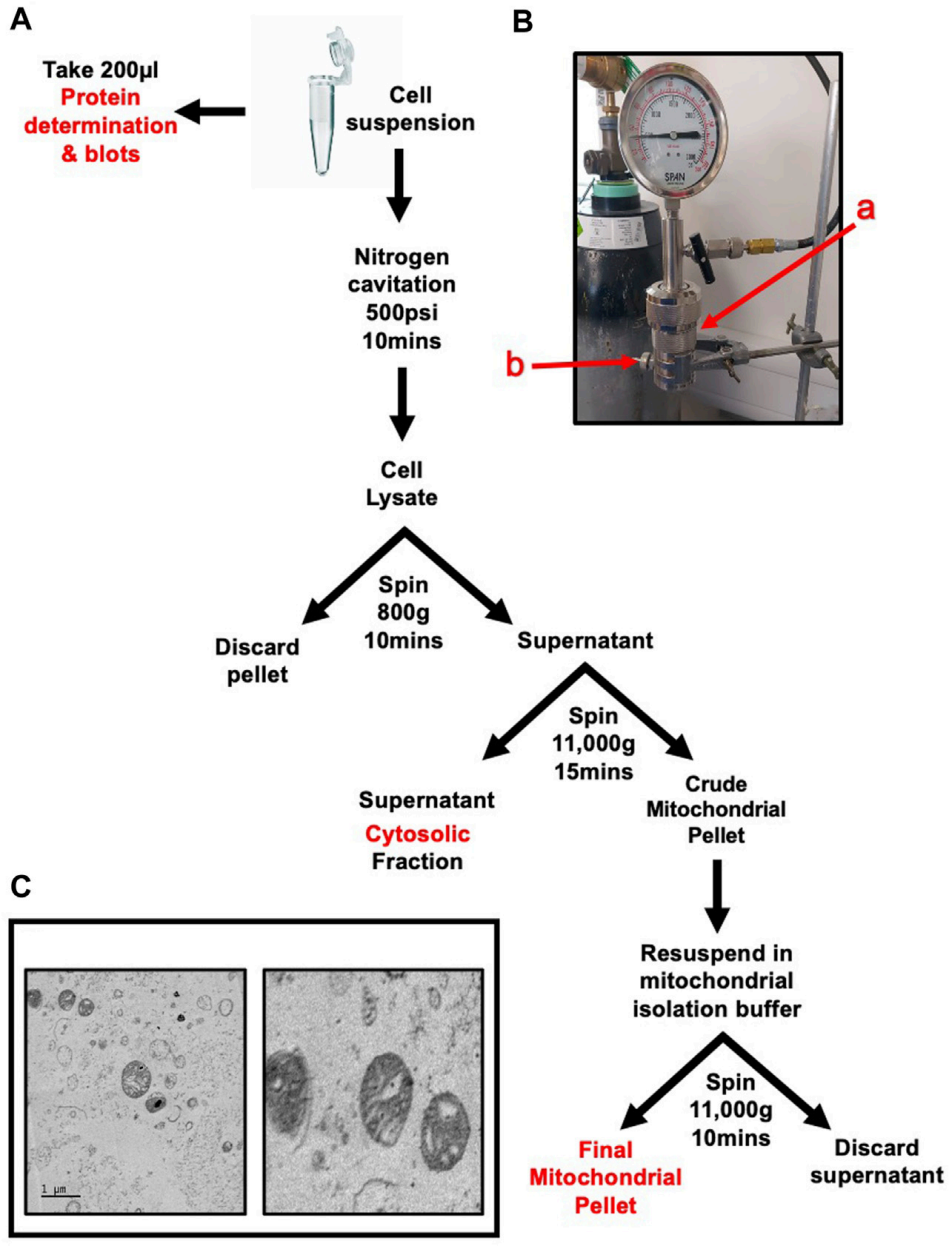
Rapid and Intact skeletal muscle mitochondrial extraction through nitrogen cavitation and differential centrifugation. Schematic of mitochondrial isolation protocol for cells or skeletal muscle tissue **(A)** Nitrogen cavitation device, label a(red) identifies the cavitation chamber, label b(red) identifies the outlet valve **(B)** Electron microscopy of recovered mitochondria at 1 µm (left) and 500 nm (right). The mitochondrial inner and outer membranes and cristae are clearly visible **(C)** The mitochondrial matrix shows no signs of damage or compromised integrity.

Cell pellets were collected by washing the monolayer twice with Dulbecco’s phosphate buffered saline without Ca^2+^ and Mg^2+^ (DPBS-) to remove traces of serum. Cells were then incubated with trypsin for approximately 1 min at 37°C. Trypsin was quenched with 10 mL of complete growth media, the suspension was then transferred to a 50 mL centrifuge tube. Cells were pelleted by centrifugation at 300 g for 5 min. The supernatant was discarded, and pellet was washed twice with PBS. The method is summarised in Figure 1A.

Once washed the pellet was resuspended in 10 mL sucrose extraction buffer (10 mM HEPES, pH 7.5, 70 mM sucrose, 200 mM mannitol, 1 mM EGTA, 1% protease inhibitors and 1% phosphatase inhibitors).

Tissue was placed in a 1.5 mL centrifuge tube in 1 mL of sucrose extraction buffer and chopped into small (approx. 2 mm) chunks. The tissue suspension was transferred into a pre-cooled Dounce homogeniser and homogenised with 10 strokes of a tight pestle. The suspension was strained sequentially through 70 µm and 40 μm cell strainers. The strained solution was made up to 10 mL with sucrose buffer.

Both cell and tissue extracts were then subject to the same steps from this point onwards. The cell suspension was placed in a nitrogen cavitation chamber (Parr Instrument Company–Cat. Number 4639) labelled a in Figure 1B) with a magnetic stirrer and was pressurised to 500psi for 10 min whilst stirring on ice. The suspension was released drop wise through the outlet valve (labelled b in Figure 1B) into a pre-chilled 15 mL tube. The cell suspension was centrifuged at 800 g for 10 min to pellet nuclei and cellular debris. The supernatant was transferred to a pre-chilled tube and the pellet discarded. The suspension was centrifuged at 11,000 g for 15 min. The resulting pellet was resuspended in 1 mL of sucrose buffer and centrifuged again at 11,000 g for 10 min to wash the mitochondrial pellet. The pellet was then resuspended in 200 µL mitochondrial assay buffer (MAS) (70 mM sucrose, 210 mM mannitol, 2 mM HEPES, 1 mM EGTA, 5 mM magnesium chloride, 10 mM potassium dihydrogen phosphate, 0.2% fatty acid-free bovine serum albumin (w/v) 10 mM sodium pyruvate, 2 mM D-Malic acid) and protein content was quantified using a modified Lowry method. Recovered mitochondria can be visualised in Figure 1C.

### Mitochondrial isolation using mechanical homogenisation-based methods

The method is provided in full by Garcia and colleagues (Garcia-Cazarin et al., 2011). Briefly, tissue was dissected rinsed in PBS and transferred to PBS + EDTA. Tissue was then transferred to isolation buffer 1 (10 mM EDTA, 215 mM D-mannitol, 600 mM sucrose, 160 mM HEPES, 0.8% w/v BSA, pH7.4) and minced with scissors. The tissue was then homogenised using a mechanical homogeniser with 10 strokes before being centrifuged for 10 min at 700 g (50 µL was taken as total homogenate for immunoblotting). The supernatant was then transferred into a fresh tube and then centrifuged at 10,500 *g* for 10 min. The resultant pellet was then resuspended in 500 µL of isolation buffer 2 (3 mM EGTA, 215 mM D-mannitol, 600 mM sucrose, 160 mM HEPES, 0.8% w/v BSA, pH 7.4) and the supernatant was collected as the ‘cyto’ fraction for immunoblotting. The suspension was once again centrifuged at 10,500 g for 10 min with the resultant pellet was the mitochondrial fraction and resuspended in 100 µL of isolation buffer 2 before the protein was quantified. For cells, the mitochondrial isolation kit for cells from Thermo Fisher (cat. 89874) was used, the manufacturer’s instructions were followed with 10 strokes of a mechanical homogeniser.

### Respirometry of isolated mitochondria

Mitochondrial respiratory studies were performed to the direction of the protocol described by (Sakamuri et al., 2018). Briefly, the SeahorseXF24 sensor cartridge was loaded with ADP 20 mM (port A), oligomycin 50 µM (port B), Carbonyl cyanide-p-trifluoromethoxyphenylhydrazone (FCCP) 50 µM (port C) and rotenone 100 µM/antimycin A 20 µM (port D). Isolated mitochondria were diluted to the desired final concentration (5 μg/50 μL) using ice-cold MAS and loaded into the wells. The cell culture microplate was centrifuged at 2,000 g for 20 min at 4°C. After centrifugation pre-warmed 150 μL of MAS was added to each well and the plate incubated in a 37°C non-CO_2_ incubator for 10 min. The plate was loaded on to the Seahorse apparatus and the injection protocol summarised in Table 1 was set up.

**TABLE 1:** Seahorse XFe24 assay protocol for mitochondria isolated from skeletal muscle cells and tissue.

Step	Duration (minutes)	Compound injected
Equilibration	12	
Mix	1	
Wait	3	
Measure	3	
Mix	1	
Wait	3	
Measure	3	
Injection A		**ADP**
6 Cycles		
Mix	1	
Measure	6	
Injection B		**Oligomycin**
6 Cycles		
Mix	1	
Measure	3	
Injection C		**FCCP**
6 Cycles		
Mix	1	
Wait	3	
Injection D		**Rotenone & Antimycin A**
6 Cycles		
Mix	1	
Measure	3	

### Western blot analysis

Cells were detached using trypsin, centrifuged at 1,200 rpm for 5 min and washed with Dulbecco’s modified Eagle’s medium. Cell pellets were weighed and resuspended in 8X pellet weight of ice-cold RIPA buffer (150 mM NaCl, 5 mM EDTA, 50 mM Tris pH 8.0, 1% NP40, 0.5% sodium deoxycholate, 0.1% sodium dodecyl sulfate). Lysates were incubated on ice for 15 min with regular vortex mixing. Protein lysates were then centrifuged at 14,000 g for 10 min at 4°C and supernatant collected. Protein content of samples was quantified using the Bio-Rad DC protein assay kit following the manufacturer’s instructions. Organelle extracts were kept in their original extraction buffer and were subject to protein content analysis as described. Equal protein aliquots per sample were subjected to electrophoresis on either a 12% or 10% sodium dodecyl sulfate-polyacrylamide gel. Separated proteins were transferred onto a nitrocellulose membrane using the Bio-Rad Trans-Blot Turbo semi-dry blotting system. Equal protein loading was assessed by staining with 0.05% copper phthalocyanine in 12 mM HCl. Blotted membranes were blocked for 1 h in 3% dried skimmed milk in Tris-buffered saline (TBS) containing 0.1% Tween-20 and incubated overnight at 4°C with primary antibodies. Cytochrome C (sc-13156), Histone H3 (sc-517576) (Santa Cruz BioTechnology), GAPDH (5174) COX IV (4850) (Cell Signalling Technology). All antibodies were used at a dilution of 1:1,000. Membranes were then washed and incubated for 2 h at room temperature with horseradish peroxidise-conjugated anti-rabbit/mouse immunoglobulin G secondary antibodies (1:1000). Antibody binding was revealed with enhanced chemiluminescence western blotting detection reagent (Thermo Fisher). Digital images were captured using a SYNGENE GBOX.

### Transmission electron microscopy

Samples were prepared according to the general procedure as described by (Kristofikova et al., 2020) but with immediate processing without storage. The resin was TAAB 813 (TAAB, Aldermaston) and after sectioning samples were stained with EM Stain 336 (Agar Scientific Ltd., Stansted) and Reynold’s stain. Once fixed, embedded, sectioned, and stained, sections were examined with a JEM2100Plus (JEOL United Kingdom, Welwyn Garden City) operating at 120 kV and operated according to the manufacturer’s procedures. Electron micrographs were digitised using a Rio16 (Gatan United Kingdom, Abingdon) camera operated using Digital Micrograph (3.32.2403.0) and exported to.tiff for further analysis.

## Results

The isolation of mitochondria through nitrogen cavitation takes approximately 45 min. Total protein yield from a 330 mg of cell pellet was 830 µg of mitochondrial protein (Table 2). This equates to 0.28% of the input protein. As mitochondria are reported to make up between 3%–5% of skeletal muscle, our method is successful in isolating nearly 30% of the cell’s mitochondria (Park et al., 2014). From tissue, the method was successful in isolating 358 µg of mitochondria from an average tissue weight of 107 mg representing 0.34% of mitochondrial recovery from total tissue input (Table 2).

**TABLE 2:** Mitochondrial protein yields.

	Input pellet weight (mg)	Mitochondrial pellet recovered ( u g)
Muscle cells	327.27 ± 13.21	847.97 ± 52.27
Muscle tissue	107.67 ± 6.69	357.79 ± 29.66

**FIGURE 2:**
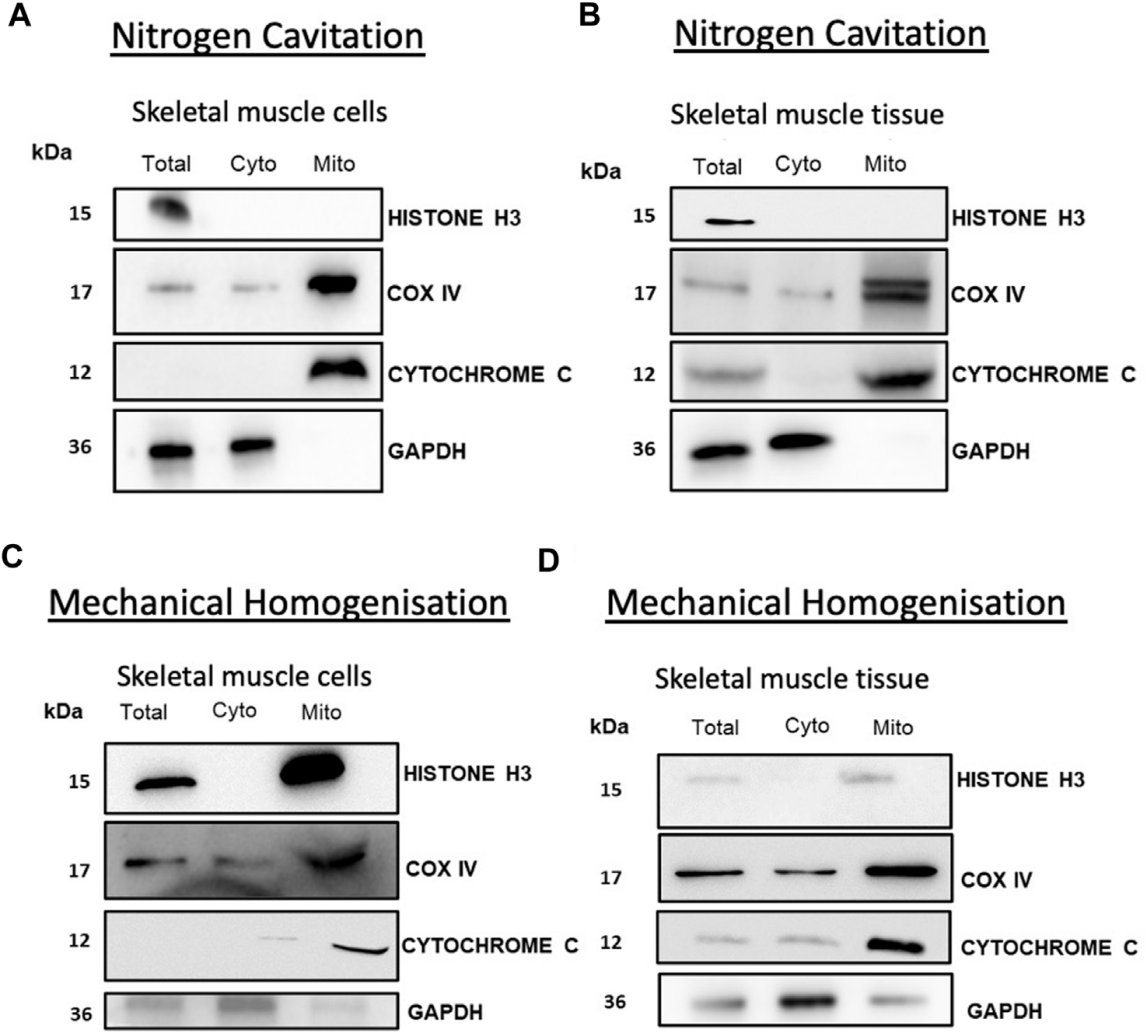
Nitrogen cavitation of skeletal muscle recovers mitochondria with reduced cytochrome C leakage. Mitochondria obtained from muscle cells **(A)** and tissue **(B)** were subject to Western blot analysis of nuclear (Histone H3) and cytosolic (GAPDH) mitochondrial fractions (cytochrome C and COX IV). Mitochondria collected from mechanical homogenisation of muscle cells **(C)** and *tibialis anterior* muscle tissue **(D)** were subject to Western blot analysis of nuclear (Histone H3) and cytosolic (GAPDH) mitochondrial fractions (cytochrome C and COX IV).

Isolated mitochondria from both tissue and cells showed no contamination from cytosolic or nuclear proteins showing a pure fraction without contamination from other major organelles. Western blot analysis of cytochrome C and COX IV showed enrichment in the mitochondrial fraction. Analysis of cytochrome C indicates that the mitochondria were intact with an absence of cytochrome C leakage into the cytosol (Figures 2A, B). To compare the efficacy of this method we compared nitrogen cavitation-based method against a widely used mechanical homogenisation-based method used to isolate mitochondria from skeletal muscle (Garcia-Cazarin et al., 2011) and a commercially available kit for cells. The results of the study show that nitrogen cavitation produced lower variability and high reproducibility in comparison to the homogenisation-based method. One of the key indicators of the success of an isolation method is the degree of purity of the mitochondrial fraction, and in this case, the mitochondrial samples generated using mechanical homogenisation were contaminated with nuclear (histone H3) and cytosolic (GAPDH) proteins (Figures 2C, D).

**FIGURE 3:**
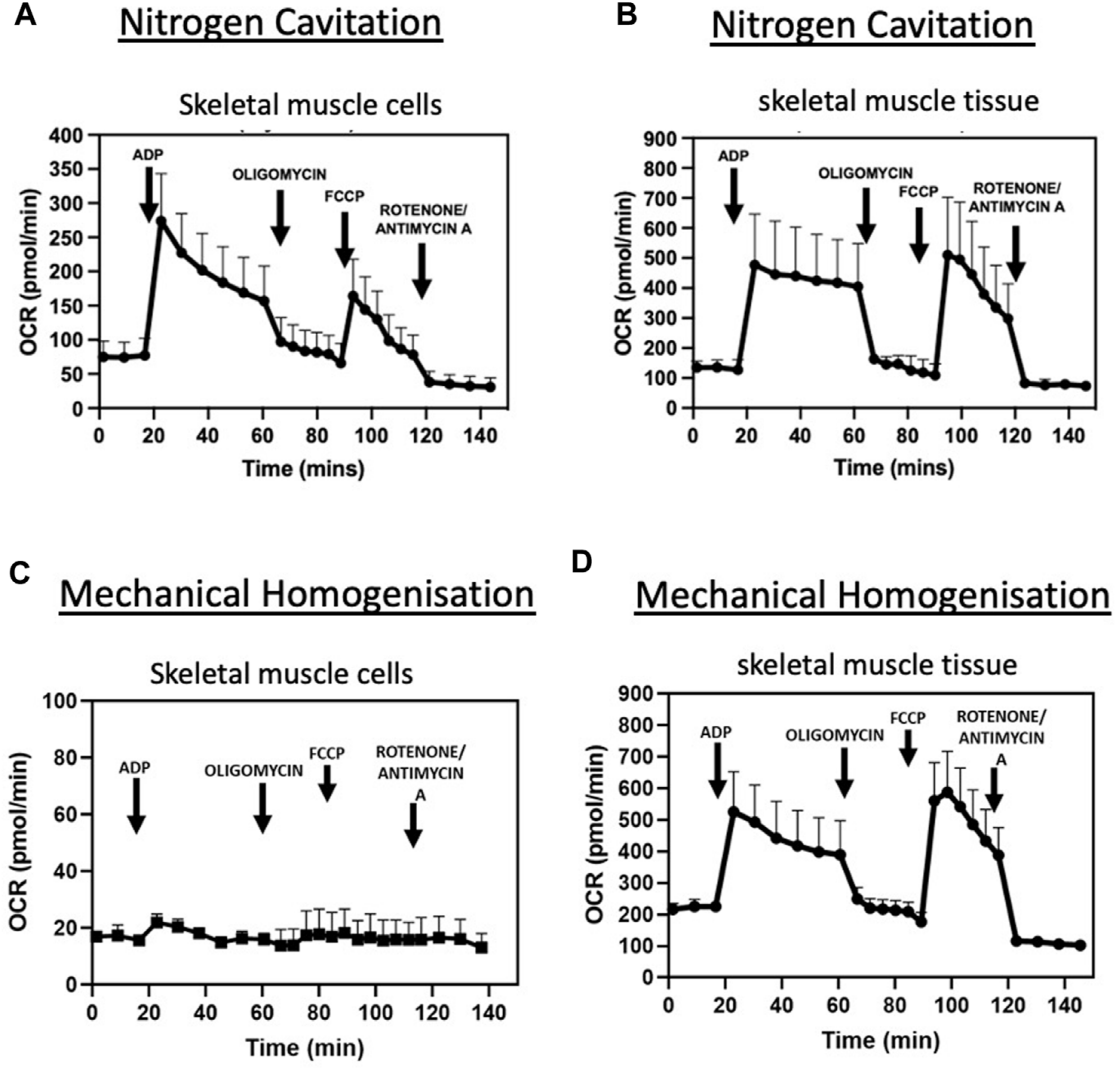
Nitrogen cavitation is successful in isolating intact, contaminant free, respiring mitochondria. OCR curves of mitochondria isolated by nitrogen cavitation for C2C12 muscle cells **(A)** and *tibialis anterior* muscle tissue **(B)** OCR curves of mitochondria obtained by mechanical homogenisation of muscle cells **(C)** and *tibialis anterior* muscle tissue **(D)** are also shown. Respiratory parameters of mitochondria in the presence of glutamate and malate (0.5M/0.5M). The rates of respiration in State 3 and in State 4 are expressed as pmol O2/min. Data represented as a mean ± S.E.M. of five different mitochondrial preparations isolated from different skeletal muscle cells and three different TA preparations. Representative plot of point-to-point OCR data in the presence of mitochondrial substrates and inhibitors. Data expressed as a mean of 5 individual replicates +SEM.

States of respiration in the isolated mitochondria from both tissue and cells were measured using a Seahorse XFe24 analyser in the presence of complex I substrates malate and pyruvate. Isolated mitochondria responded to complex I, III and V inhibitors (Figures 3A, B). These data demonstrate the nitrogen cavitation method was able to preserve mitochondrial respiratory activity. The respiratory control ratio of isolated mitochondria from C2C12 myoblast cells was 4.9 ± 0.8 in the presence of malate and glutamate and for skeletal muscle tissue was 4.6 ± 0.2. State III respiration was measured at an oxygen consumption rate (OCR) of 273.8 ± 69.20 pmol/min and state IV respiration measured at an average OCR of 71.4 pmol/min ±26.70 for mitochondria isolated from cells. For mitochondria isolated from skeletal muscle tissue, state III respiration was measured at an OCR of 480.27 ± 174.87 pmol/min and state IV at 107.72 ± 39.78 pmol/min (Table 3).

**TABLE 3:** Respiration states of isolated mitochondria from nitrogen cavitation.

	State III (pmol/min)	State IV (pmol/min)	Respiratory control ratio
Muscle cells	273.80 ± 69.20	71.40 ± 26.70	4.90 ± 0.80
Muscle tissue	480.27 ± 174.87	107.72 ± 39.78	4.55 ± 0.17

Moreover, states of respiration in the isolated mitochondria from mechanical homogenisation-based methods were measured using a Seahorse XFe24 analyser in the presence of complex I substrates malate and pyruvate and responded to complex I, III and V inhibitors (Figures 3C, D). Though the respiratory control ratio, a measure of the efficiency of the mitochondrial electron transport chain, was found to be lower at 2.89 ± 0.21 for the tissue and 1.59 ± 0.7 for cells (Table 4) whilst the RCR from nitrogen cavitation was 4.55 and 4.9. This shows the mechanical homogenization-based method may also disrupt the structural integrity of the mitochondria, reducing in their functional capacity. Finally, the total yield of mitochondria was also a lot lower in the mechanical homogenisation-based method with only 104 µg of mitochondria recovered from an initial tissue weight of 166 mg (Table 5) representing a recovery of just under 0.1% compared to 0.34% from nitrogen cavitation. The mitochondrial recovery was also lower from cells with 55 µg of mitochondria recovered from an initial pellet weight of 294 mg representing a percentage recovery of 0.01% (Table 5).

**TABLE 4:** Respiration states of isolated mitochondria from mechanical homogenisation-based method.

	State III (pmol/min)	State IV (pmol/min)	Respiratory control ratio
Muscle cells	22.1 ± 2.9	13.9 ± 5.6	1.59 ± 0.7
Muscle tissue	525.42 ± 110.17	176.92 ± 25.32	2.89 ± 0.21

**TABLE 5:** Mitochondrial protein yield from mechanical homogenisation-based method.

	Input pellet weight (mg)	Mitochondrial pellet recovered ( u g)
Muscle cells	295.2 ± 12.6	55.7 ± 3.4
Muscle tissue	166.25 ± 13.91	104.51 ± 8.97

## Discussion

The selection of a mitochondrial extraction protocol is done to best suit the demands of the upstream experimental workflow and downstream endpoints. Herein, we provide an additional protocol for the rapid and robust isolation of mitochondria. This would be ideal for studies of a larger scale or those requiring higher throughput respirometry analysis.

Direct comparisons between different methodologies are challenging due to inherent variations such as cell type, chemical reagents, equipment and methodological disparities. However, utilised protocols for mitochondrial isolation each have their own limitations (Sims and Anderson, 2008; Hornig-Do et al., 2009; Djafarzadeh and Jakob, 2017).

Our method has the benefits of consistent cellular lysis followed by brief differential centrifugation to isolate intact, respiring mitochondria with a comparable RCR at the top end of the range stated in the literature (Lombardi et al., 2000; Hornig-Do et al., 2009; Vuda et al., 2012; Djafarzadeh and Jakob, 2017). As expected, and evident in other methods, lysosomal and endoplasmic reticulum (ER) contamination are apparent using our method. However, there are no nuclear, cytoplasmic or whole-cell contaminants (Figures 2A, B). As there are several contact sites between mitochondria and the ER it is not unexpected that there is some ER present (Supplementary Figure S1) (Lee and Min, 2018; Moltedo et al., 2019; Xia et al., 2019). In this regard, our method for isolating mitochondria may hold significant advantages for mitochondrial proteomic analysis. Due to technological advances of LC-MS/MS and the availability of up-to-date and in-depth databases including MitoCarta (Rath et al., 2021), it may be unnecessary to have ultrapure mitochondrial fractions as proteins can be matched to these databases and other proteins from contaminants, such as lysosomes, filtered out. Therefore, we present a valuable alternative to existing mitochondrial isolation protocols. This protocol is applicable to tissue and cells of skeletal muscle origin and will be of potential use to those working with *in vivo* primary endpoints that have downstream *in vitro* mitochondrial evaluation.

## Data Availability

The original contributions presented in the study are included in the article/Supplementary Materials, further inquiries can be directed to the corresponding author.

## Supplementary material

SUPPLEMENTARY FIGURE S1 ER and Lysosomal contaminants in mitochondrial fraction. Electron microscope image of mitochondrial fraction at 1 µm, with red arrows indicating endoplasmic reticulum and blue arrows indication lysosomes **(A)** Western blot analysis of fractions showing existence of ER contaminants in mitochondrial fractions recovered from cells **(B)** and muscle tissue **(C)**.

## Abbreviations

OCR, Oxygen consumption rate; MAS, mitochondrial assay buffer; FCCP, Carbonyl cyanide-p-trifluoromethoxyphenylhydrazone; TBS, Tris-buffered saline; RCR, Respiratory control ratio.
