# Supplementary material for: Rapid isolation of respiring skeletal muscle mitochondria using nitrogen cavitation
Source: Front Physiol. 2023 Mar 7;14:1114595. doi: 10.3389/fphys.2023.1114595 (PMC10027933; doi:10.3389/fphys.2023.1114595)
Supplement: Supplementary file 1 [file Presentation1.pdf]

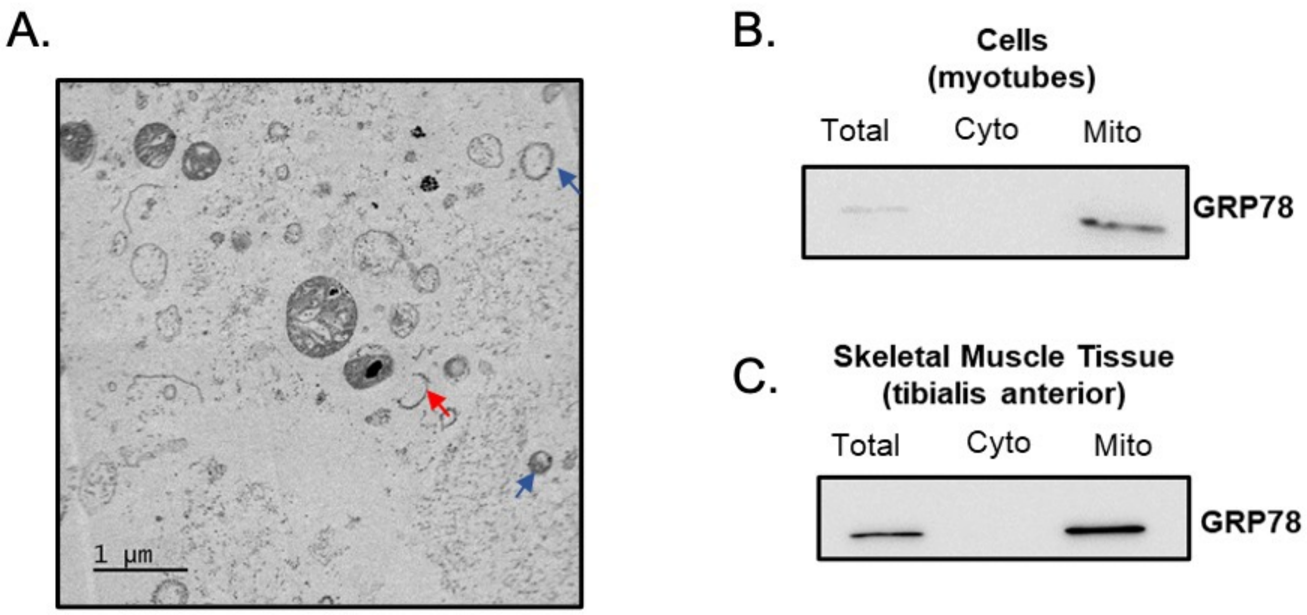

Supplementary figure 1. ER and Lysosomal contaminants in mitochondrial fraction. Electron microscope image of mitochondrial fraction at 1μm, with red arrows indicating endoplasmic reticulum and blue arrows indication lysosomes (A). Western blot analysis of fractions showing existence of ER contaminants in mitochondrial fractions recovered from cells (B) and muscle tissue (C).
